# Estimating the NIH Efficient Frontier

**DOI:** 10.1371/journal.pone.0034569

**Published:** 2012-05-02

**Authors:** Dimitrios Bisias, Andrew W. Lo, James F. Watkins

**Affiliations:** 1 Sloan School of Management, Massachusetts Institute of Technology, Cambridge, Massachusetts, United States of America; 2 Computer Science and Artificial Intelligence Laboratory and Department of Electrical Engineering and Computer Science, Massachusetts Institute of Technology, Cambridge, Massachusetts, United States of America; 3 AlphaSimplex Group, LLC, Cambridge, Massachusetts, United States of America; 4 Department of Surgery, Brigham and Women’s Hospital and Harvard Medical School, Boston, Massachusetts, United States of America; Tel Aviv University, Israel

## Abstract

**Background:**

The National Institutes of Health (NIH) is among the world’s largest investors in biomedical research, with a mandate to: “…lengthen life, and reduce the burdens of illness and disability.” Its funding decisions have been criticized as insufficiently focused on disease burden. We hypothesize that modern portfolio theory can create a closer link between basic research and outcome, and offer insight into basic-science related improvements in public health. We propose portfolio theory as a systematic framework for making biomedical funding allocation decisions–one that is directly tied to the risk/reward trade-off of burden-of-disease outcomes.

**Methods and Findings:**

Using data from 1965 to 2007, we provide estimates of the NIH “efficient frontier”, the set of funding allocations across 7 groups of disease-oriented NIH institutes that yield the greatest expected return on investment for a given level of risk, where return on investment is measured by subsequent impact on U.S. years of life lost (YLL). The results suggest that NIH may be actively managing its research risk, given that the volatility of its current allocation is 17% less than that of an equal-allocation portfolio with similar expected returns. The estimated efficient frontier suggests that further improvements in expected return (89% to 119% vs. current) or reduction in risk (22% to 35% vs. current) are available holding risk or expected return, respectively, constant, and that 28% to 89% greater decrease in average years-of-life-lost per unit risk may be achievable. However, these results also reflect the imprecision of YLL as a measure of disease burden, the noisy statistical link between basic research and YLL, and other known limitations of portfolio theory itself.

**Conclusions:**

Our analysis is intended to serve as a proof-of-concept and starting point for applying quantitative methods to allocating biomedical research funding that are objective, systematic, transparent, repeatable, and expressly designed to reduce the burden of disease. By approaching funding decisions in a more analytical fashion, it may be possible to improve their ultimate outcomes while reducing unintended consequences.

## Introduction

The National Institutes of Health (NIH) is among the world’s largest and most important investors in biomedical research. Its stated mission is to “seek fundamental knowledge about the nature and behavior of living systems and the application of that knowledge to enhance health, lengthen life, and reduce the burdens of illness and disability” (http://www.nih.gov/about/mission.htm). Some have criticized the NIH funding process as not being sufficiently focused on disease burden [1]–[3] (further discussion about criticisms and recommendations for improving the process of allocating research funds is provided in Text S1 and Table S1). Even after allowing for extensive private-sector translational investment, significant funding gaps between disease states persist [4]. Furthermore, carefully considered changes in funding may generate dynamic effects that create more “mouths to feed” [5]. NIH leaders have observed that research is risky, involving trade-offs among stated criteria, and also unstated secondary objectives, e.g., actively “balancing out” spending by other entities [6]. These factors pose challenges to allocating research funds.

We consider a framework in which biomedical research allocation decisions are more directly tied to the risk/reward trade-off of burden-of-disease outcomes. Prioritizing research efforts is analogous to managing an investment portfolio: in both cases, there are competing opportunities to invest limited resources, and expected returns, risk, correlations, and the cost of lost opportunities are important factors in determining the return of those investments.

Financial decisions are commonly made according to portfolio theory [7], in which the optimal trade-off between risk and reward among a collection of competing investments–known as the “efficient frontier”–is constructed via quadratic optimization, and a point on this frontier is selected based on an investor’s risk/reward preferences. Given a measure of “return on investment” (ROI), an “efficient portfolio” is defined to be the investment allocation that yields the highest expected return for a given and fixed level of risk (as measured by return volatility), and the locus of efficient portfolios across all levels of risk is the efficient frontier.

We recast the NIH funding allocation decision as a portfolio-optimization problem in which the objective is to allocate a fixed amount of funds across a set of disease groups to maximize the expected “return on investment” (ROI) for a given level of volatility. We define ROI as the subsequent improvements in years of life lost (YLL), and using historical time series data provided by the NIH (http://www.nih.gov/about/almanac/appropriations/index.htm) and the Centers for Disease Control (CDC, http://wonder.cdc.gov/) for each of 7 disease groups, we estimate the means, variances, and covariances among these time series. These estimates serve as inputs to the portfolio-optimization problem. Such an approach provides objective, systematic, transparent, and repeatable metrics that can incorporate “real-world” constraints, and yields well-defined optimal risk-sensitive biomedical research funding allocations expressly designed to reduce the burden of disease.

Portfolio theory highlights the value of diversification: investing in multiple securities with imperfectly correlated pay-offs almost always yields a better reward-to-risk profile than investing in a single security. For developing this framework, Markowitz shared the Nobel Memorial Prize in Economic Sciences, and today portfolio theory is the starting point for investment management decisions among the largest institutional investors [8], [9]. Recently, portfolio theory has also been proposed as a means for conducting health care cost-benefit analysis [10]–[12].

## Methods

### Funding Data

**Figure 1:**
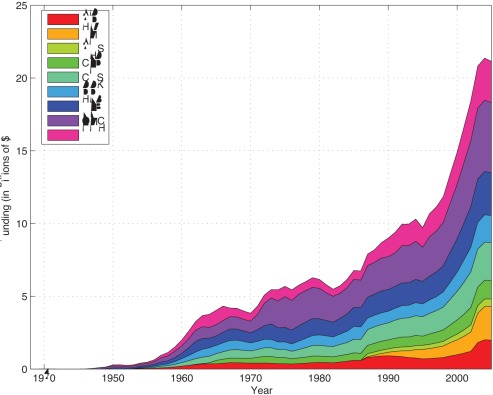
Appropriations data. NIH appropriations in real (2005) dollars, categorized by disease group (http://www.nih.gov/about/budget.htm).

The NIH has 27 Institutes and Centers, of which we identified 10 with research missions clearly tied to specific disease states, and which account for $21 billion of funding in 2005 or 74% of the total. The disease classification scheme used and the procedure for constructing the appropriation time series are described in greater detail in Table S2 and Figure S1. Figure 1 depicts NIH appropriations data in real (2005) dollars from 1965 to 2005, and summary statistics are provided in Table S3. The National Institute of Allergies and Infectious Diseases (NIAID) spending has been split to account for HIV, which is presented separately (see HIV discussion below).

These Institutes and the basic research they fund have inevitable overlap and effect beyond their charter; we treat all spending for any given Institute as being directed toward the corresponding disease states, and account for spillover effects by considering the correlations in the lessening of the burden of disease in other groups. For example, molecular biology funded by the NCI may be relevant to infectious diseases but, like the entire NCI budget, would be assumed for modeling purposes to be directed at cancer; the hypothetical infectious-disease improvement would appear in the correlation between the decrease in years of life lost for cancer and that of infectious diseases.

### Burden of Disease Data

Because of its simplicity, availability, breadth, and long history, years of life lost (YLL) was chosen as the measure of burden of disease to be used in constructing the estimated return on investment from NIH-funded research (see Text S1 for a discussion of other possible measures). The CDC Wide-ranging Online Data for Epidemiologic Research (WONDER) database (http://wonder.cdc.gov/) was queried for the underlying cause of death at the Chapter level (except for mental disorders, where dementia and unspecified psychoses were all placed in CNS for consistency with CDC coding after 1998) for International Classification of Diseases (ICD) categories ICD-9 (for 1979–1998) and ICD-10 (for 1999–2007). The two datasets for pre- and post-1998 were joined into one continuous series, data were stratified into groups by age at death, and YLL were computed by comparing the midpoint of the age ranges with the World Health Organization’s (WHO) year-2000 U.S. life table (http://www.who.int/whosis/en/). Years of life lost were then tabulated by Chapter annually, and adjusted for population growth to remove what would otherwise be a systematic downward bias in realized health improvements. This process yielded YLL series for 9 distinct disease groups (see Table S2 for an analysis of HIV separated from other infectious disease).

**Figure 2:**
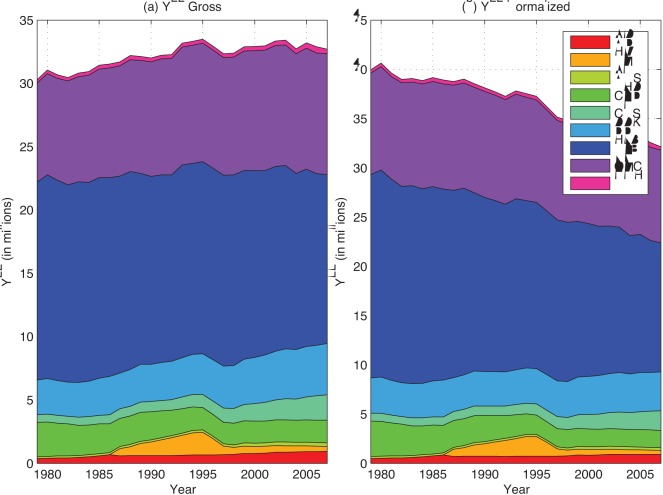
YLL data. Panel (a): Raw YLL categorized by disease group (http://wonder.cdc.gov/). Panel (b): Population-normalized YLL (with base year of 2005), categorized by disease group. Both panels are based on data from 1979 to 2007.

Using 2005 as the base year, the raw YLL observations were adjusted in other years to be comparable to the 2005 population:
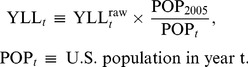
(1)The procedure for assembling the YLL time series is summarized in Figure S2, and the resulting series, both raw and normalized for population growth, are shown in Figure 2. Summary statistics for these adjusted YLL series are also reported in Table S3 for the 9 groups (HIV treated separately). The change in burden of disease was measured by taking first differences. These first differences were used to compute the “return on investment” on which the mean-variance optimizations were based (see the “Methods” section below).

Three disease areas required special consideration: HIV, AMS, and dementia. AMS and HIV have shorter histories, which is problematic for estimating parameters based on historical returns that are lagged by typical FDA approval times plus 4 years. Dementia, including Alzheimer’s disease and unspecified psychoses, was reclassified with the change from ICD-9 to ICD-10 from mental and behavioral disorders to diseases of the nervous system; we placed all dementia YLL in the CNS group to avoid a transition-point artifact at the juncture between ICD-9 and ICD-10, and then performed a sensitivity analysis with and without the dementia YLL. Further work could, in a manner analogous to our treatment of HIV, treat this group of neurodegenerative conditions as a separate category.

HIV poses a special challenge given its extreme returns after the introduction of protease inhibitors, which are outliers that are likely to be non-stationary and would heavily bias the parameter estimates on which the portfolio optimization is based. To address this outlier, HIV spending and its corresponding YLL were omitted from those of other infectious diseases–the component of NIAID spending directed at HIV was estimated by straight-line interpolation from published figures, and this HIV spending was treated as a separate entity and subtracted from reported NIAID appropriations; a similar procedure was followed for the estimation of HIV-related YLL, and WONDER was queried at the subchapter level to implement this separation.

For completeness, empirical results that include AMS and HIV data are provided in Text S1, but because of their unique characteristics, these two groups are omitted from our main empirical results other than the summary tables.

### Applying Portfolio Theory

To apply portfolio theory, the concept of a “return on investment” (ROI) must first be defined. Although YLL has already been chosen as the metric by which the impact of research funding is to be gauged, there are at least two issues in determining the relation between research expenditures and YLL that must be considered. The first is whether or not any relation exists between the two quantities. While the objectives of pure science do not always include practical applications that impact YLL, the fact that part of the NIH mission is to “reduce the burdens of illness and disability” suggests the presumption–at least by the NIH–that there is indeed a non-trivial relation between NIH-funded research and burden of disease. For the purposes of this study, and as a first approximation, we assume that YLL improvements are proportional to research expenditures. Of course, factors other than NIH research expenditures also affect YLL, including research from other domestic and international medical centers and institutes, spending in the pharmaceutical and biotechnology industries, public health policy, behavioral patterns, prosperity level and environmental conditions. Therefore, the YLL/NIH-funding relation is likely to be noisy, with confounding effects that may not be easily disentangled. See the Discussion section above for a more detailed discussion of this assumption and some possible alternatives.

The second issue is the significant time lag between research expenditures and observable impact on YLL. For example, Mosteller [13] cites a lag of 264 years, starting in 1601, for the adoption of citrus to prevent scurvy by the British merchant marine. More contemporary examples [14]–[16] cite lags of 17 to 20 years. We use shorter lags in this study both because of data limitations (our entire dataset spans only 29 years), and also to reduce the impact of factors other than research expenditures on our measure of burden of disease (YLL). Any attempt to optimize appropriations to achieve YLL-related objectives must take this lag into account, otherwise the resulting optimized appropriations may not have the intended effects on subsequent YLL outcomes.

**Figure 3:**
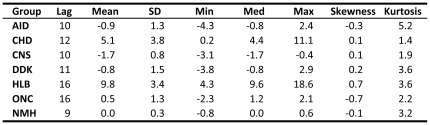
Return summary statistics. Summary statistics for the ROI of disease groups, in units of years (for the lag length) and per-capita-GDP-denominated reductions in YLL between years *t* and *t*+4 per dollar of research funding in year *t*–*q*, based on historical ROI from 1980 to 2003.

The impact of NIH-funded research on disease burden is likely to be spread out over several years after this intervening lag, given the diffusion-like process in which research results are shared in the scientific community. For simplicity, the same duration (*p* = 5 years) of the diffusion-like impact for all the disease groups was hypothesized. The lag *q* for each disease group was estimated by running linear regressions associating improvements in YLL over *p* = 5 years with NIH funding *q* years earlier and real income and choosing the lag between 9 and 16 years (beyond which data limitations and other factors make it impossible to distinguish the impact of research funding from other confounding factors affecting YLL) that maximizes the *R* ^2^ and the corresponding lags are shown in Figure 3.

This procedure is, of course, a crude but systematic heuristic for relating research funding to YLL outcomes. Alternatives include using a single fixed lag across all groups, simply assuming particular values for group-specific lags based on NIH mandates and experience, computing a time-weighted average YLL for each group with a weighting scheme corresponding to an assumed or estimated knowledge-diffusion rate for that group, or constructing a more accurate YLL return series by tracking individual NIH grants within each group to determine the specific impact on YLL (through new drugs, protocols, and other improvements in morbidity and mortality) from the award dates to the present. While the choice of lag is critical in determining the characteristics of the YLL return series and deserves further research, it does not effect the applicability of the overall analytical framework. While our procedure is surely imperfect, it is a plausible starting point from which improvements can be made.

Assuming constant impact of research funding on YLL over the duration of *p* years, the measure of the ROI that accrues to funds allocated in year *t* is then given by:
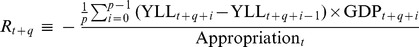
(2)


where the minus sign reflects the focus on *decreases* in YLL, and the multiplier 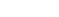 is per capita real gross domestic product (GDP) in year *t*+*q* (http://www.bea.gov/national/index.htm#personal), which is included to convert the numerator to a dollar-denominated quantity to match the denominator. This ratio’s units are then comparable to those of typical investment returns: date- (*t*+*q*) dollars of return per date-*t* dollars of investment.

Given the definition in equation (2) for the ROI of each of the disease groups, the “optimal” appropriation of funds among those groups must be determined, i.e., the appropriation that produces the best possible aggregate expected return on total research funding per unit risk. Denote by 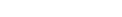 the vector of returns of all *n* groups for a given appropriation date *t* (where time subscripts have been suppressed for notational simplicity), and denote by *µ* and Σ the vector of expected returns and the covariance matrix, respectively. If the weights of the budget allocation among the groups are 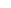, the ROI for the entire portfolio of grants, denoted by *R_p_*, is given by 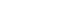, and its expected value and variance are 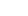 and 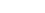, respectively. The objective function to be optimized is then given by the expected value minus some multiple of the variance which reflects risk tolerance, and this quadratic function of 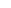 is maximized using standard quadratic optimization techniques (see Text S1), subject to the constraint that the weights sum to 1.

In some financial applications, a variation of this optimization problem is employed in which the objective function is augmented to include penalty for allocations that deviate from some pre-specified vector of target weights 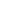 such as a “benchmark” portfolio. Accordingly, we also consider a “dual-objective” optimization problem in which the additional penalty term is proportional to deviations from existing NIH allocations 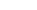, and the proportionality constant is given by the coefficient 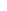. When 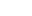, the augmented objective function reduces to the single-objective function described above; when it is positive, the dual-objective function will yield optimal weights that are closer to 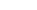.

## Results

### Summary Statistics

Summary statistics of the ROI for the period 1980–2003 are presented in Figure 3. An example of the ROI calculation for HLB for 1986 when the return was 18.6 is given in Table S4, summary statistics of the ROI with AMS and HIV groups included are given in Table S5, and the correlation matrix of the ROIs is given in Table S7. Large differences in mean ROI for different Institutes are evident in Figure 3, ranging from small negative values ( *e.g.*, –1.7 for CNS) to large positive values ( *e.g.*, 9.8 for HLB). Large differences in standard deviation also exist, ranging from 0.3 for NMH to 3.8 for CHD, implying important risk/reward tradeoffs across Institutes.

**Figure 4:**
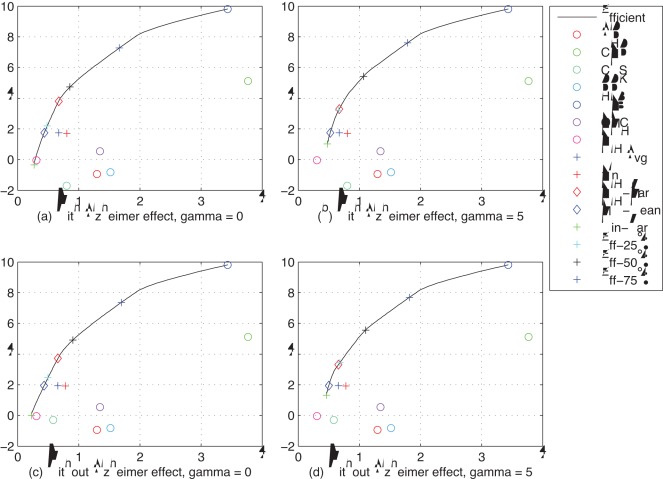
Efficient frontiers. Efficient frontiers for (a) all groups except HIV and AMS, 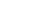; (b) all groups except HIV and AMS, 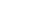; (c) all groups except HIV and AMS without the dementia effect, 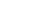; and (d) all groups except HIV and AMS without the dementia effect, 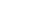; based on historical ROI from 1980 to 2003. The region labeled “DP” indicates portfolios that dominate the historical average NIH portfolio.

Negative mean ROIs are counterintuitive–implying that increasing investment is counterproductive to easing the burden of disease–yet they occur in three disease groups: AID, CNS, and DDK. There are several reasons for this phenomenon. First, and foremost, unlike investments in financial assets, there is significant randomness in the relation between NIH spending and subsequent impact on YLL. Many factors other than the amount of funding affect the success or failure of pure and translational research, and average ROI values reflect the impact of all of those factors. For example, in the case of CNS–in which the negative return is more than two standard deviations away from 0–there is a large non-stationary effect due to the rapid growth of a group of dementias. A sensitivity analysis confirms that the negative returns are largely due to the dementia effect as is indicated in sub-panels (c) and (d) of Figure 4.

A more subtle effect comes from the fact that favorable ROIs in one area can impart negative bias in other groups, since all deaths must be assigned to one cause or group. Consider a simple thought experiment in which only two lethal diseases, A and B, exist. If a cure for A is discovered, then those who would otherwise have died of A must necessarily die of B eventually. This yields an increase in the YLL for B, even if the treatment of B diseases has not worsened. Similar, if less-extreme, dynamics can emerge with more disease groups and less-dramatic progress that merely reduces rather than eliminates the YLL burden of a specific disease group.

Finally, and perhaps least likely, the dissemination of erroneous research results [17] could, in principle, increase YLL until the error is identified and remedied.

Rather than “correcting” these counterintuitive empirical relations, we view them as important anomalies that deserve further scrutiny and analysis. In some cases, e.g., CNS, the anomaly can be traced to a specific external factor that can either be accepted as legitimate or set aside as an extreme outlier that is not representative of the true relation between funding and subsequent YLL. In the latter case, one alternative to using an empirically estimated mean ROI to compute the efficient frontier is to impose a Bayesian prior on this parameter (see Text S1 for details).

To develop intuition for possible patterns between funding allocation and improvements in YLL, the cumulative sums of these two variables are plotted in Figure S5 and the eigenvalues and eigenvectors of the estimated covariance matrices are provided in Figure S6. These results suggest that dimension-reducing strategies such as linear factor models may be useful in this domain. However, without a more detailed understanding of the common drivers of progress (if any) among the groups, dimension reduction via principal components or factor analysis may yield misleading results due to overfitting.

### Efficient Frontiers

In Figure 4, efficient frontiers for the single- and dual-objective (see equation 2 in Text S1) optimization problems are plotted in mean-standard deviation space for the 7-group cases with and without taking into account the dementia effect. Figure S4 depicts the corresponding 9-group frontiers. For each of these frontiers, in addition to the mean-standard deviation points for the different disease groups, the corresponding points for the following funding allocations are also plotted:

- historical average NIH allocation for years 1996–2005;
- equal-weighted (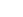) allocation;
- minimum-variance allocation;
- the allocation on the efficient frontier that has the same mean as the average NIH allocation (the “NIH-mean” allocation);
- the allocation on the efficient frontier which has the same variance as the average NIH allocation (the “NIH-var” allocation);
- the allocation on the efficient frontier that is 25% of the distance from the minimum variance allocation to the maximum expected-return allocation;
- the allocation on the efficient frontier that is 50% of the distance from the minimum variance allocation to the maximum expected-return allocation;
- the allocation on the efficient frontier that is 75% of the distance from the minimum variance allocation to the maximum expected-return allocation.

**Figure 5:**
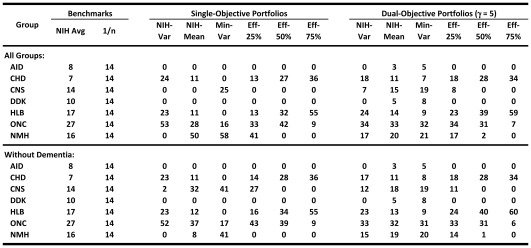
Portfolio weights. Benchmark, single- and dual-objective optimal portfolio weights (in percent), based on historical ROI from 1980 to 2003.

**Figure 6:**
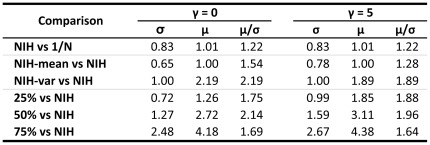
Relative performance. Relative volatility (σ), expected return (µ) and risk-adjusted returns (µ/σ) for different scenarios (see text) for both 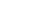 and 5 compared, including dementia: NIH with uniform allocation, and scenarios with NIH historical performance. A value of 1.00 implies the same performance, 0.92 implies 8% worse, while 1.12 implies 12% improvement.

The region bounded by the horizontal segment (*i*–*iv*), the vertical segment (*i*–*v*), and the efficient frontier (marked “DP”) is of special interest because all portfolios in this region offer lower variance, higher expected return, or both when compared to the average NIH allocation, hence from a mean-variance perspective such allocations are unambiguously preferable. These allocations are called “dominating” portfolios relative to the average NIH allocation (*i*). Figure 5 contains the portfolio weights corresponding to portfolios depicted in Figure 4, and Figure 6 provides a relative-performance comparison of these portfolios in terms of their expected returns, volatilities, and ratios of the two.

Figure 4A shows that a number of the disease groups appear to be concentrated in a relatively low-risk sector of the risk/reward universe, which may be evidence of active variance-minimization strategies by various stakeholders. Further, Figure 6 suggests that NIH appropriations are lower volatility than the 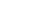 portfolio, while returns are maintained.

A sensitivity analysis is conducted by estimating the efficient frontier with (Figure 4A) and without the dementia effect (Figure 4C), and the upper and lower panels of Figure 5 contains the portfolio weights to these two cases.

The top left sub-panel of Figure 5 shows that the single-objective optimization does yield sparse weights as expected. For example, the minimum-variance portfolio allocates to only three groups: 58% to NMH, 25% to CNS, and 16% to ONC. By minimizing variance, irrespective of the mean, this portfolio allocates funding to groups with least variability in YLL improvements. The efficient-25% portfolio allocates non-zero weights in four groups (41% to NMH, 33% to ONC, 13% to HLB, and 13% to CHD), and yields 26% better expected return with 28% less risk (Figure 6). With still more emphasis on expected return, the efficient-50% portfolio gives non-zero weights only to three successful groups: 42% to ONC, 32% to the higher risk, higher expected-return HLB, and 27% to CHD. This portfolio has 172% higher expected return but only 27% more risk than the NIH portfolio. The efficient-75% portfolio gives an even higher weight of 55% to HLB, 36% to CHD, and 9% to ONC, yielding 318% higher expected return and 148% more risk, a diminishing risk-adjusted expected return as compared to portfolios with lower volatility. Given the greater emphasis on expected return for this portfolio, it is not surprising to see HLB getting a bigger role due to its apparent historical success in reducing YLL. Of course, whether or not past success is indicative of comparable future success hinges on the science and associated translational efforts underlying the diseases covered by HLB. This underscores the importance of incorporating research and clinical insights into the funding allocation process, especially within a systematic framework such as portfolio theory.

However, the dementia effect may underestimate the performance of the CNS disease group, hence the lower panel of Figure 5 reports corresponding optimal-portfolio results without the dementia effect. In the single-objective case, the efficient-50% and 75% portfolios are still sparse, with non-zero weights in 3 groups, while the lower risk efficient-25% portfolio is less concentrated with non-zero weights to 4 groups and significant weight (27%) to the CNS group.

Figure 5 also contains the optimal portfolios for the dual-objective case (with 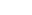) in the right sub-panels (see Figure 4B and 4D). These cases correspond to portfolios that trade off closeness to the average NIH allocation policy with better risk-adjusted expected returns. Now we observe that for both upper and lower sub-panels corresponding to the 7-group with/without the dementia effect optimization, respectively, the weights are less concentrated than in the single-objective case. For example, the minimum-variance portfolio without the dementia effect now allocates funding to all the groups, with weights ranging from 5% to 31%. However, even in this case, the efficient-75% portfolio is still extreme, allocating weights only to HLB, CHD and ONC. Therefore, special care must be exercised in selecting the appropriate point on the efficient frontier. We also observe from the NIH-var or NIH-mean portfolios that slight changes to the average NIH policy apparently yield superior performance in mean-standard deviation space (Figure 6 indicates 28% to 89% relative improvement, depending on the assumptions). Table S6 contains the portfolio weights when the HIV and AMS groups are included in our analysis.

## Discussion

Portfolio theory is a systematic framework for determining optimal research funding allocations based on historical return on investment, variance, and correlation between appropriations and reductions in disease burden. The optimization results suggest that significant YLL improvements with respect to a mean-variance criterion may be possible through funding re-allocation. To our knowledge, this is the first time such an approach has been empirically implemented in this domain.

Our method identifies optimal portfolios which, for a given degree of risk, are efficient in reducing YLL. However, some optimal allocations may allocate no funds to certain disease groups (typically those with low expected return and high volatility). While this may be reasonable from a mean-variance optimization perspective, it is obviously an extreme and impractical outcome. A first step toward recognizing the trade-off between reallocation costs and efficiency is the dual-objective optimization procedure which penalizes allocations away from a pre-specified “benchmark” allocation, e.g., the current NIH policy. This more-conservative approach increases diversification with less reallocation cost than the single-objective procedure. For instance, in the dual-objective optimization, the NIH-mean portfolio is relatively close to the current allocation (see Figure 5), yet the risk-adjusted returns are 28% higher as seen in Figure 6. The method is also sufficiently flexible to add further constraints reflecting other policy goals such as imposing lower or upper bounds on expenditures for disease groups.

Our findings must be qualified in at least three respects: (1) YLL as a measure of burden of disease, which is clearly incomplete and less than ideal; (2) the definition of ROI and the challenges of relating research expenditures to subsequent outcomes such as burden of disease; and (3) the known limitations of portfolio theory from the financial context. Each of these qualifications is discussed in greater detail in Text S1, and while they can all be addressed to varying degrees through additional data and analysis, the empirical conclusions are likely to depend critically on the nature of their resolution. In this section, we provide a short synopsis of these qualifications, and also consider other objections to this framework and directions for future research.

YLL captures only the most extreme form of disease burden; more refined measures such as disability-adjusted or quality-adjusted life years are clearly preferable. However, time series histories for such measures are currently unavailable. Therefore, YLL is the most natural starting point for gauging the impact of biomedical research funding, and is directly aligned with the NIH mission to “lengthen life”.

Our definition of ROI can also be challenged as being imprecise and *ad hoc* in several respects. NIH funding is typically focused on basic research rather than translational efforts, therefore, NIH spending may not be as directly related to subsequent YLL improvements. We have not accounted for other expenditures that may also affect YLL, and to the extent that NIH appropriations are systematically used to complement private spending [6], the relation between NIH allocations and YLL improvements may be even noisier. Also, the standard portfolio-optimization framework implicitly assumes a constant multiplicative relation between dollars invested today and dollars returned tomorrow (so that doubling the investment will typically double the ROI of that investment), whereas the return to biomedical investments may be non-linear and will likely exhibit diminishing returns. Furthermore, returns are bounded–the best we can hope for is a cure, beyond which further improvement is impossible. In addition, translational research takes time and significant non-NIH resources, further blurring the relation between NIH allocations and subsequent changes in YLL. Finally, other factors may contribute to YLL improvements, including changes in cultural norms, economic conditions, and public policy.

While all of these qualifications have merit, they are not insurmountable obstacles and can likely be addressed through additional data collection and more sophisticated metrics, perhaps along the lines of Porter [18] or Lane and Bertuzzi [19]. Moreover, the portfolio-optimization approach provides a useful conceptual framework for formulating funding allocation decisions systematically, even if its empirical implications are imprecise.

There are also several limitations of portfolio theory that are well-known in the financial context (e.g., estimation error, parameter instability, and exogenous constraints such as non-negativity restrictions on portfolio weight), all of which can be addressed to some degree through statistical techniques such as resampling, Bayesian analysis, and robust optimization [20]. However, one limitation that is unique to biomedical applications is the fact that portfolio theory is silent on which mean-variance-optimal portfolio to select. In the financial context, the existence of a riskless investment (e.g., U.S. Treasury bills) implies that one unique portfolio on the efficient frontier will be desired by all investors–the so-called “tangency” portfolio [21] (see Figure S3). Because there is no analog to a riskless investment in biomedical research, the notion of a tangency portfolio does not exist in this context. Therefore, decision makers must first determine society’s collective preferences for risk and return with respect to changes in YLL before a unique solution to the portfolio-optimization problem can be obtained, i.e., they must agree on a societal “utility function” for trading off the risks and rewards of biomedical research.

This critical step is a pre-requisite to any formal analysis of funding allocation decisions, and underscores the need for integration of basic science with biomedical investment performance analysis and science policy. Such integration will require close and ongoing collaboration between scientists and policymakers to determine the appropriate parameters for the funding allocation process, and to incorporate prior information and qualitative judgments [22] regarding likely research successes, social priorities, policy objectives and constraints, and hidden correlations due to non-linear dependencies not captured by the data. In particular, it is easy to imagine contexts in which funding objectives can and should change quickly in response to new environmental threats or public-policy concerns. However, such pressing needs must be balanced against the disruptions–which can be severe due to the significant adjustment costs implicit in biomedical research [23]–caused by large unanticipated positive or negative shifts in research funding. Although the end result of collaborative discussion may fall short of a well-defined objective function that yields a clear-cut optimal portfolio allocation, the portfolio-optimization process provides a transparent and rational starting point for such discussions, from which several insights regarding the complex relation between research funding and social outcomes are likely to emerge.

These qualifications suggest that portfolio theory cannot be mechanically applied to historical data to yield actionable optimal allocations. However, our empirical results should be sufficient proof-of-concept to motivate additional data collection, empirical analysis, and research to advance the state of the art in this application area. Any repeatable and transparent process for making funding allocation decisions–especially one that involves criteria other than peer-review-based academic excellence–will, understandably, be viewed initially with some degree of suspicion and contempt by the scientific community. But if one of the goals of biomedical research is to reduce the burden of disease, some tension between academics and public policy may be unavoidable. Moreover, in the absence of a common framework for evaluating the trade-offs between academic excellence and therapeutic potential, other proposed alternatives such as political earmarking [24] may be even less palatable from scientific and humanitarian perspectives.

In an environment of tightening budgets and increasing oversight of appropriations, portfolio theory offers scientists, policymakers, and regulators–all of whom are, in effect, research portfolio managers–a rational, systematic, transparent, and reproducible framework in which to explicitly balance expected benefits against potential risks while accounting for correlation among multiple research agendas and real-world constraints in allocating scarce resources. Most funding agencies and scientists have already been making such trade-offs informally and heuristically. There may be additional benefits to making such decisions within an explicit framework based on standardized and objective metrics.

One of the most significant benefits from adopting such a framework may be the reduction of uncertainty surrounding future funding-allocation decisions. This alone would greatly enhance the ability of funding agencies and scientists to plan for the future and better manage their respective budgets, research agendas, and careers. By approaching funding decisions in a more analytical fashion, it may be possible to improve their ultimate outcomes while reducing the chances of unintended consequences.

## Supporting Information

Figure S1 **NIH time series flowchart.** Flowchart for the construction of NIH appropriations time series. “NIH Approp.” denotes NIH appropriations; “PHS Gaps” denotes Institute funding by the U.S. Public Health Service; “Complete Approp.” denotes the union of these two series; “FY Change” allows for the change in government fiscal years; “4Q FY” time series refers to the resulting series in which all years are treated as having four quarters of three months each; and “CPI” refers to the Consumer Price Index.
(EPS)

Figure S2 **YLL time series flowchart.** Flowchart for the construction of years of life lost (YLL) time series. “WONDER Chapter Age Group” refers to a query to the CDC WONDER database at the chapter level, stratified by age group at death; “US Pop.” is the United States population from census data as expressed in the WONDER dataset; and “US GDP” denotes U.S. gross domestic product.
(EPS)

Figure S3
**Efficient frontier illustration.** Efficient frontier (blue) in mean-standard deviation space, indifference curves (green), and optimal portfolio *T* which is the tangency point of the efficient frontier and the highest indifference curve achievable (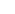) by a frontier portfolio. Point *A* corresponds to the minimum-variance portfolio.(EPS)

Figure S4
**Empirical estimates of NIH efficient frontiers.** Efficient frontiers for (a) all groups, 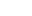; (b) all groups, 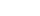; (c) all groups without the Alzheimer effect, 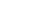; and (d) all groups without the Alzheimer effect, 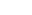; based on historical ROI from 1980 to 2003, except for AMS ROI which is available only from 1997 to 2007, and HIV ROI which is available only from 1996 to 2007.(EPS)

Figure S5 **Learning curves.** Each of the seven graphs shows for each of the disease groups (labeled at the top of each graph), a curve representing the cumulative spending in millions of dollars along the horizontal axis and the future cumulative change in millions of years of life lost (YLL) on the vertical, where the offset *q* between current spending and future changes in YLL is given in Figure 3. The cumulative change in YLL covers the years from 1980 to 2007 for all the disease groups. For each group, the corresponding NIH appropriations are translated back *q* years.
(EPS)

Figure S6 **Eigenvalues and eigenvectors.** Cumulative eigenvalues and eigenvectors of the sample covariance matrix of annual returns of the 7 NIH groups, based on historical returns from 1980 to 2003.
(EPS)

Table S1 **IoM recommendations.** 12 major recommendations of the 1998 Institute of Medicine panel in four large areas for improving the process of allocating research funds.
(EPS)

Table S2 **ICD mapping.** Classification of ICD-9 (1978–1998) and ICD-10 (1999–2007) Chapters and NIH appropriations by Institute and Center to 7 disease groups: oncology (ONC); heart lung and blood (HLB); digestive, renal and endocrine (DDK); central nervous system and sensory (CNS) into which we placed dementia and unspecified psychoses to create comparable series as there was a clear, ongoing migration noted from NMH to CNS after the change to ICD-10 in 1999; psychiatric and substance abuse (NMH); infectious disease, subdivided into estimated HIV (HIV) and other (AID); maternal, fetal, congenital and pediatric (CHD). The categories LAB and EXT are omitted from our analysis.
(EPS)

Table S3 **Summary statistics for YLL and funding.** Summary statistics for YLL and NIH-appropriations time series data. YLL data are from 1979 to 2007, except for HIV YLL data which are only available from 1987 to 2007. NIH-appropriations data are from 1965 to 2005, except for AMS and HIV appropriations data which are only available from 1987 to 2005.
(EPS)

Table S4 **ROI example.** An example of the ROI calculation for HLB from 1986.
(EPS)

Table S5 **Summary statistics for ROI.** Summary statistics for the ROI of disease groups, in units of years (for lag length) and per-capita-GDP-denominated reductions in YLL between years *t* and *t*+4 per dollar of research funding in year *t*–q, based on historical ROI from 1980 to 2003, except for AMS ROI which is available only from 1997 to 2007, and HIV ROI which is available only from 1996 to 2007.
(EPS)

Table S6 **Portfolio weights for all groups.** Single- and dual-objective optimal portfolio weights (in percent), based on historical ROI from 1980 to 2003, except for AMS which is available only from 1997 to 2007, and HIV which is available only from 1996 to 2007.
(EPS)

Table S7 **Seven-group correlation matrix.** Correlation matrix of ROI (in percent), based on historical ROI time series from 1980 to 2003.
(EPS)

Text S1 **Supporting Information.** NIH background and literature review, details of data construction and analysis, the analytics of traditional and Bayesian portfolio optimization, additional empirical results, and limitations and qualifications of our analysis are included in this file.
(PDF)
