# Supplementary material for: Estimating the NIH Efficient Frontier
Source: PLoS One. 2012 May 2;7(5):e34569. doi: 10.1371/journal.pone.0034569 (PMC3342295; doi:10.1371/journal.pone.0034569)
Supplement: Text S1 — Supporting Information. NIH background and literature review, details of data construction and analysis, the analytics of traditional and Bayesian portfolio optimization, additional empirical results, and limitations and qualifications of our analysis are included in this file. (PDF) [file pone.0034569.s014.pdf]

Estimating the NIH Efficient Frontier: Supporting Information

Dimitrios Bisias[1], Andrew W. Lo[1,2,3,*], James F. Watkins[4]
1 MIT Sloan School of Management, 100 Main Street Cambridge, MA 02142, USA
2 MIT CSAIL and EECS, 32 Vassar Street Cambridge, MA 02139, USA
3 AlphaSimplex Group, LLC, 1 Cambridge Center, Cambridge, MA 02142, USA
4 Department of Surgery, Brigham and Women's Hospital and Harvard Medical School, 75 Francis St., Boston, MA 02115, USA
∗ E-mail: alo@mit.edu

1 NIH Background and Literature Review

The National Institutes of Health (NIH) was established in 1938 and has a budget of over $31 billion, of which 80% is awarded in competitive research grants to more than 325,000 researchers through nearly 50,000 competitive grants at over 3,000 universities, medical schools, and other research institutions (http://www.nih.gov/about/budget.htm). The NIH allocates funding among competing priorities by assessing such priorities with respect to five major criteria [1]: (a) public needs; (b) scientific quality of the research; (c) potential for scientific progress (the existence of promising pathways and qualified investigators); (d) portfolio diversification; and (e) adequate support of infrastructure (human capital, equipment, instrumentation, and facilities). This framework was supported, with some additional recommendations, by an Institute of Medicine (IoM) blue-ribbon panel in 1998 (see Table S1) [2].

Despite this framework and the IoM endorsement, NIH funding has been criticized as not being aligned to disease burden and insufficiently effective [3–5]. For example, the impact of cancer has been estimated as only 5% of total direct cost but 23% of all deaths [6], while extramural spending by the National Cancer Institute (NCI) is about 15% of the total (http://report.nih.gov/). Sandler, *et al.* [7] suggested that digestive diseases were relatively underfunded based on comparisons of disease burden as measured by direct and indirect cost. Gross [8] noted that NIH funding is reasonably predicted by some burden-of-disease metrics (disability-adjusted life-years or DALY, which are unavailable in time-series form) [9]. Earmarks or target funding levels for specific diseases and programs have been suggested by a number of policymakers [10]. Differences between current and projected disease burden complicate allocation decisions, as in the growth phase of the AIDS epidemic [11].

Funding allocation decisions are not unique to the NIH; in a study similar to Gross *et al.* [8], Curry *et al.* [12] has questioned the allocations of the Centers for Disease Control. Some have argued that the academic centres conducting research are ill-suited to encourage adoption of research results [13]. Even after allowing for extensive private-sector translational investment, significant funding gaps between disease states—as measured by new-drug approvals—exist [14]. The mismatch between per-capita healthcare spending and outcome in the OECD countries lead Crow [15] to conclude that "What is missing is not 'translational' research, but stronger links between all types of knowledge-generating activities related to health, and a focus on outcomes beyond science". Teitelbaum noted the structural difficulties posed by reallocating funding leading to a larger pool of researchers [16]. Also, social medicine investigators have described gaps between researchers' agendas and those of patients [17]. NIH leaders have noted that funding basic research is itself a risky endeavour, involving trade-offs among all five of their funding criteria, and may also include unstated secondary objectives, e.g., actively "balancing out" spending by other agencies, charities, and the private sector [18]. Collectively, these factors impose significant challenges to determining an ideal allocation of research funds.

Although the economic impact of biomedical research has been considered [19], the main focus has been

on measuring value-added rather than determining optimal funding allocations. Murphy and Topel [20] estimate U.S. economic surplus from improved health on the order of $2.6 trillion annually, with benefits distributed unequally across age and gender, and suggest that in some cases, incremental benefits may not exceed the cost of achieving them. Johnston *et al.* [21] found a return to society in the form of averted treatment costs and public health benefits divided by cost of trial expenditures of 46% for clinical trials at the National Institute of Neurological Disorders and Stroke (NINDS), where the returns or net savings were generated by four of the 28 trials examined, and collectively exceeded the costs of not only the clinical, but the entire program of research at NINDS during the study period. Cutler and McClellan [22] computed returns of technological advances for five conditions and found net benefit for four and costs equal to benefits in the fifth. Buxton *et al.* [19] suggested that the most appropriate measures of the value of health care research are those that accurately identify valuable research, ascribe the impact of such research, and value the impact, ideally in economic terms. Wooding *et al.* [23] used payback techniques for 16 case studies and valued projects along five ordinal axes, noting wide variation in research payback. Fleurence and Togerson [24] suggested that research should be allocated to provide the most health benefits to the population, subject to equity considerations, and observed that subjective, burden-of-disease, and payback methods all failed this test to some degree. Instead, they argue that a method of information valuation is superior.

Modern financial portfolio theory—in which the expected return, risk (as measured by volatility), and correlations of a collection of investment opportunities are taken as inputs, and the set of all portfolio weights with the highest expected return for a given level of risk is the output—produces rational allocations of limited resources among competing priorities. For developing this method in 1952, Markowitz shared the Nobel Memorial Prize in Economic Sciences in 1990. The theory has had extensive applications among mutual funds, pension funds, endowments, and sovereign wealth funds [25–27].

More recently, portfolio theory has been proposed as a means for conducting risk-sensitive cost-benefit analysis for health-care budgeting decisions [28–33]. The motivation for these studies is the observation that typical cost-effectiveness studies of healthcare programs ignore the uncertainty of realized costs, which can be addressed by applying portfolio theory to balance the risks against the rewards of specific budget allocations. These studies present simplified frameworks for incorporating risk into the healthcare budgeting process, e.g., two-security examples (although [32] does contain 11 hypothetical cost/effect distributions) and do not contain full-scale empirical applications to realistic budgeting tasks. As the authors note, applying portfolio theory to large public healthcare reimbursement problems can be challenging. Patients may have differing and non-constant utility functions, and some argue that the manager/administrator should only consider expected returns, allowing the patient and physician to consider risk trade-offs at individual treatment levels, in which case the aggregate utility function (see Figure S3) is implicit. In such an arrangement, if new treatments are more expensive, have incremental yield, and a higher variance, we note the possibility of a net migration or "risk-ratcheting" toward high-cost, high-risk therapies, a scenario of obvious interest. In a single-payer system, the administrator has little ability to address portfolio composition if the portfolio is considered to be the entire patient population. However, the method can be applied to a fixed population faced with competing risk/return/treatment combinations for a given disease. This line of inquiry is focused on cost reimbursement, not on estimating return on research for which the problem of stochastic return is more complex and degree of administrator freedom possibly greater.

Despite the growing interest in measuring the return on biomedical research [34, 35], and the fact that portfolio theory has already been applied to healthcare budgeting decisions, some sceptics continue to argue against the use of any quantitative metrics in this domain. For example, Black [36] states categorically that "[t]he biomedical 'payback' approach is certainly inappropriate and attempts to impose it should be strenuously resisted. Instead, a qualitative approach should be applied that takes into account the 'slow-burning fuse' and avoids simple attribution of cause and effect". While such a response may be acceptable for certain types of funding, it is becoming increasingly untenable with respect to public

funds and government support, which, by law, almost always require some form of cost/benefit analysis, performance attribution, and oversight.

2 Data Issues

Measures of Disease Burden. As a measure of disease burden, YLL captures only lethal illness by definition; chronic illness enters the optimization process only indirectly, mortality in the young is more heavily weighted than that of the elderly, and quality-of-life is not captured at all. The choice of YLL is motivated by several factors: long time-series observations of YLL are readily available, they cover a large population, and they address the entire spectrum of diagnoses categorized under the ICD (see http://www.who.int/classifications/icd/en/). Broader measures of burden of disease such as disability adjusted life years (DALY) [8] and quality-adjusted life years (QALY) [37, 38] have been proposed, but historical time series for such measures are not yet available. As better measures are developed (*e.g.*, incidence, prevalence, physician visits, hospitalization, DALY, QALY), portfolio-optimization methods may be applied to them as well through appropriately defined "returns". Should datasets covering not only age and cause of death but also ante-mortem symptoms become available, mean-variance-efficient allocations would likely place significant weight on improvements in the care of less-lethal chronic diseases.

Definition of Return on Investment. Even if YLL is an appropriate measure of disease burden, the corresponding construction of the ROI to NIH funding is a noisy estimate of the impact of research dollars on improvements in YLL in several respects. First, NIH funding typically supports basic research and a few clinical trials, not the subsequent translational efforts required to prevent disease and affect practice. Therefore, the relation between NIH spending and changes in YLL is not nearly as direct as the relation between investing in the stock market and changes in one's net worth. Moreover, to the extent that NIH appropriations are systematically used to complement private spending to allocate total funding across diseases more fairly [18], the relation between NIH funding and subsequent YLL improvements may be even noisier, and may require modelling private-sector expenditures as a separate but complementary portfolio-optimization problem with an objective function and constraints that are linked to those of the NIH.

Second, the standard portfolio-optimization framework implicitly assumes a multiplicative relation between dollars invested today and dollars returned tomorrow. Moreover, the multiplier is constant and unaffected by the amount of the investment, so that doubling the investment will typically double the ROI of that investment. However, in the case of NIH funding allocations, it is not at all clear that doubling the budget of a given Institute will double its impact on future changes in YLL—the output of translational medicine is not necessarily linear in its funding costs. In fact, a doubling of NIH expenditures did occur between 1998 and 2003, when its annual spending grew from $14 billion to $27 billion, and while it is still too early to assess the full impact of this increase, Couzin and Miller [39] and Freeman and van Reenen [40] have observed a number of disruptive effects of this sudden increase and equally sudden decline in funding. The fact that scientific research programs cannot be initiated or dismantled quickly implies non-linearities in the relation between expenditures and even the simplest measures of research output, e.g., the number of journal publications. In fact, the NIH has stated that appropriations are partly allocated to "balance out" the effects of other entities' activities [18], which can create complex non-linearities in the relation between NIH allocation decisions and future changes in YLL.

Third, the fact that translational research takes time and significant non-NIH resources implies additional noise in associating funding decisions in year $t-q$ with improvements in YLL in years t through $t+4$. Moreover, improvements in YLL may be due to unrelated factors such as changes in cultural norms (including consumption of alcohol and cigarettes), economic conditions (such as recessions vs. expansions), and public policy (such as vaccine programs and mandates for automobile, home, and workplace safety).

The estimates of q were an initial attempt to link appropriation with outcome in a systemic and non-discretionary manner, but they were derived heuristically from regulatory, appropriation, and epidemiological data which may not be stationary or predictive. For example, if the Food and Drug Administration's capacity for reviewing new-drug applications is held constant and applications double, substantial increases in regulatory queuing would be expected, even with the added resources generated by the Prescription Drug User Fee Act (PDUFA, see http://www.fda.gov/ForIndustry/UserFees/PrescriptionDrugUserFee/default.htm).

Finally, in converting changes in YLL to dollar amounts, per-capita real GDP was used as the "conversion factor" irrespective of age, despite the fact that children and retired individuals are economically less active.

While these caveats highlight the imprecision with which the impact of research spending is measured, they also provide direction for developing better metrics. In particular, the underlying science of each grant implies a particular set of dynamics for translation and YLL impact, and with more sophisticated models of such dynamics, the returns to fundamental research should be measurable with greater accuracy.

3 Mean-Variance Portfolio Optimization

In the mean-variance framework, we seek to find the best trade-offs between risk and expected return by varying the portfolio weights $\boldsymbol{\omega}$ to trace out the locus of mean-variance combinations that cannot be improved upon, i.e., that are "efficient". This set of efficient portfolios, also known as the "efficient frontier" is formally defined as the curve in mean-variance (or mean-standard deviation) space corresponding to all portfolios with the highest level of expected return for a given level of variance (the blue curve in Figure S3). This efficient frontier defines the set of allocations that cannot be improved upon from a mean-variance perspective, and the optimal allocation is a single point on this frontier that is determined by the investor's desired volatility level or risk tolerance. More formally, an investor with standard mean-variance preferences is assumed to prefer portfolios with greater expected return and lower variance, with diminishing returns in each (so that progressively greater increments of expected return must be offered to the investor to induce him to accept increases in the same increment of risk as the level of risk rises). This type of preferences generates so-called "indifference curves" (non-intersecting curves in mean-variance space that trace out combinations of mean and variance for which an individual is indifferent) that are upward sloping and convex (see Figure S3). The optimal portfolio for a given set of indifference curves is the tangency point T of the efficient frontier with the most upper-left indifference curve (curve U_1 in Figure S3).

To compute the efficient frontier, the following optimization problem must be solved (we maintain the following notational conventions: (1) all vectors are column vectors unless otherwise indicated; (2) matrix transposes are indicated by a prime superscript, hence $\boldsymbol{\omega}'$ is the transpose of $\boldsymbol{\omega}$; and (3) vectors and matrices are always typeset in boldface, i.e., X and μ are scalars and $\mathbf{X}$ and $\boldsymbol{\mu}$ are vectors or matrices):

$$\begin{aligned} &\underset{\boldsymbol{\omega}}{\text{Minimize}} && \boldsymbol{\omega}'\boldsymbol{\Sigma}\boldsymbol{\omega} \\ &\text{subject to} && \boldsymbol{\omega}'\boldsymbol{\mu} \geq \mu_o \ \ , \ \ \boldsymbol{\omega}'\boldsymbol{\iota} = 1 \ \ , \ \ \boldsymbol{\omega} \geq 0 \end{aligned} \tag{S1}$$

where $\boldsymbol{\iota}$ is an $(n \times 1)$-vector of 1's and μ_o is an arbitrary fixed level of expected return. By varying μ_o between a range of values and solving the optimization problem for each value, all the efficient allocations $\boldsymbol{\omega}^*$ may be tabulated, and the locus of points in mean-standard-deviation space corresponding to these efficient allocations is the efficient frontier depicted in blue in Figure S3 . This so-called Markowitz portfolio optimization problem involves minimizing a quadratic objective function with linear constraints, which is a standard quadratic programming (QP) problem that can easily be solved analytically in some cases [41], and numerically in all other cases by a variety of efficient and stable solvers [42, 43].

One additional refinement to address the well-known issue of "corner solutions" (in which several components of $\boldsymbol{\omega}^*$ are 0) that often arise in the standard portfolio-optimization framework is proposed. While such extreme allocations may, indeed, be optimal with respect to the mean-variance criterion, they are more often the result of estimation error and outliers in the data [44]. Moreover, even in the absence of estimation error, mean-variance optimality may not adequately reflect other objectives such as social equity across disease groups or distance from current status quo in allocation. To incorporate such considerations, a "regularization" technique is applied in which the objective function is penalized for allocations that are far away from the average allocation policy. Specifically, consider the following regularized version of the standard portfolio-optimization problem:

$$\begin{aligned} \underset{\boldsymbol{\omega}}{\text{Minimize}} \quad & \boldsymbol{\omega}'\boldsymbol{\Sigma}\boldsymbol{\omega} \ + \ \gamma \left\| \boldsymbol{\omega} - \boldsymbol{\omega}_{NIH} \right\|^2 \\ \text{subject to} \quad & \boldsymbol{\omega}'\boldsymbol{\mu} \geq \mu_o \ , \ \boldsymbol{\omega}'\boldsymbol{\iota} = 1 \ , \ \boldsymbol{\omega} \geq 0 \end{aligned} \tag{S2}$$

This formulation is essentially a dual-objective optimization problem in which the first objective is to minimize the portfolio's variance ($\boldsymbol{\omega}'\boldsymbol{\Sigma}\boldsymbol{\omega}$), and the second objective is to minimize the difference from the average NIH allocation policy ($\|\boldsymbol{\omega} - \boldsymbol{\omega}_{NIH}\|^2$) and the non-negative parameter γ determines the relative importance of these two objectives. Larger values of γ yield optimal weights that are closer to average NIH allocation but which correspond to portfolios with greater volatility, and smaller values of γ yield optimal weights that may be more concentrated among a smaller subset of groups, but which imply lower portfolio volatility.

4 Results Including HIV and AMS

Since HIV and AMS time series have shorter histories, we set $p=1$ for these groups and calculate their lags by the same method as for the other groups. The corresponding lags are shown in Table S5.

Summary Statistics. Summary statistics of the ROI for HIV based on years 1996–2007 (Table S5) show that HIV presents a notable instance of non-stationary behaviour. Its mean ROI of 90.8 is an order of magnitude larger than the second-best-performing group and its standard deviation of 167.1 is 50 times larger than the group with the second-largest standard deviation.

Efficient Frontiers. In Figure S4 , the efficient frontiers for the single- and dual-objective optimization problems are plotted in mean-standard-deviation space for the 9-group cases with and without the dementia effect. For each of these frontiers, the mean-standard-deviation points for the same set of portfolios as in the main paper are also plotted.

Figure S4 a shows that the distribution of ROI of the HIV group is skewed, and very sensitive to effects of several years of remarkable out-performance immediately after the introduction of protease inhibitors. Therefore, HIV yields an influential or "high-leverage" set of data points with estimated means and volatilities that are outliers.

The top left sub-panel of Table S6 shows that the single-objective optimization does yield sparse weights as expected. For example, the minimum-variance portfolio allocates to only three groups: 75% to NMH, 15% to CNS, and 10% to ONC. By minimizing variance, irrespective of the mean, this portfolio allocates funding to groups with least variability in YLL improvements. The efficient-25% portfolio is even less diversified, with non-zero weights in only two groups: 84% to HLB and 16% to HIV. As in the case with seven groups, it is not surprising to see HLB playing a much bigger role given its apparent historical success in reducing YLL and the greater emphasis on expected return for this portfolio.

With still more emphasis on expected return, the efficient-50% portfolio also gives non-zero weights only to these two successful groups: 57% to HLB and 43% to the higher risk, higher mean-return HIV.

The efficient-75% portfolio gives even higher weight to HIV, 72% and 28% to HLB. However, as discussed above, the historical performance of HIV is an outlier that may not be representative of this group's future impact on YLL. In addition, the dementia effect may underestimate the performance of CNS disease group, hence the lower panel of Table S6 reports corresponding optimal-portfolio results without the dementia effect. In the single-objective case, the efficient-25, 50% and 75% portfolios are almost unchanged given the historical outperformance of HIV, while the lower risk minimum variance portfolio is also concentrated to 3 groups but with much more significant weight 26% to the CNS group.

Table S6 also contains the optimal portfolios for the dual-objective case (with $\gamma = 10$) in the right sub-panels (see Figures S4b and S4d). These cases correspond to portfolios that trade off closeness to the average NIH allocation policy with better risk-adjusted rewards. Now we observe that for both upper and lower sub-panels corresponding to the 9-group with/without the dementia effect optimization, respectively, the weights are less concentrated than in the single-objective case. For example, the minimum-variance portfolio without the dementia effect portfolio now allocates funding to 7 groups, with weights ranging from 7% to 28%. However, even in this case, the efficient-75% portfolio is still extreme, allocating weights only to HIV and HLB. Therefore, special care must be exercised in selecting the appropriate point on the efficient frontier.

5 Bayesian Portfolio Optimization

Let $\boldsymbol{\Sigma}_o$ be a symmetric matrix, where the (i, j) component is the experts' estimation of the covariance between the returns of the corresponding disease groups. Then, we can find its projection in the convex cone of positive semi-definite matrices, that is the positive semi-definite matrix $\bar{\boldsymbol{\Sigma}}$, whose distance from $\boldsymbol{\Sigma}_o$ (defined as the sum of the squares of the differences of the corresponding components of the matrices) is minimized. Then we can formulate the following regularized optimization problem:

$$
\begin{aligned}
\underset{\boldsymbol{\omega}}{\text{Minimize}} \quad & \boldsymbol{\omega}'\boldsymbol{\Sigma}\boldsymbol{\omega} \;+\; \gamma\boldsymbol{\omega}'\bar{\boldsymbol{\Sigma}}\boldsymbol{\omega} \\
\text{subject to} \quad & \boldsymbol{\omega}'\boldsymbol{\mu} \geq \mu_o \;\;,\;\; \boldsymbol{\omega}'\boldsymbol{\iota} = 1 \;\;,\;\; \boldsymbol{\omega} \geq 0 \;.
\end{aligned}
$$

Higher moments of returns (beyond mean and variance) and different measures of risk could also be accommodated [45]. In contrast to financial markets, we believe that it is reasonable to assume that the forecast correlation and covariance is comparatively stable over time, and that exceptions to this belief, such as epidemics, should be reasonably identifiable.

6 Summary Statistics

To develop intuition for possible patterns between funding allocation and improvements in YLL, the cumulative sums of these two variables are plotted in Figure S5 . Notable findings include a sustained improvement in cardiovascular and lung (HLB) mortality and a long period of relative under-performance in oncology (ONC) followed by a turnaround.

The eigenvalues and eigenvectors of the estimated covariance matrices, summarized in Figure S6, suggest that dimension-reducing strategies such as linear factor models may be useful in this domain. However, without a more detailed understanding of the common drivers of progress (if any) among the groups, dimension reduction via principal components or factor analysis may yield misleading results due to overfitting.

7 Limitations of Portfolio Theory

Even within the exact domain for which it was developed, portfolio theory has several well-known limitations, of which the most obvious is the possibility that the mean-variance criterion may not, in fact, be the appropriate objective function to be optimized. While there is little disagreement that higher expected ROI is preferable to its alternative, the trade-off between expected ROI and risk is fraught with subtleties involving specific psychological, perceptual, and behavioural mechanisms of individuals and groups. Because of these considerations, mean-variance analysis is often considered an approximation to a much more complicated reality—a starting point for investment allocation decisions, not the final answer.

Another known limitation of portfolio theory is the fact that the input parameters $(\boldsymbol{\mu}, \boldsymbol{\Sigma})$ must be estimated from historical data, and estimation error in these parameter estimates can lead to portfolios that are unstable and sub-optimal [25]. One common approach to addressing this problem in the financial context is to employ prior information regarding the input parameters, thereby reducing the dependence on historical data. Using Bayesian methods, expert opinions regarding the statistical properties of the individual asset returns can be incorporated into the portfolio optimization process [46, 47] (see also the discussion of Bayesian portfolio optimization above).

References

1. National Institutes of Health (1997) Setting Research Priorities at the National Institutes of Health. Bethesda, MD: National Institutes of Health.

2. Committee on the NIH Research Priority-Setting Process, *Scientific Opportunities and Public Needs: Improving Priority Setting and Public Input at the National Institutes of Health*, pp. 11–12. Washington, D.C.: National Academy Press. (1998).

3. Rangel SJ, Efron B, Moss RL (2002) Recent trends in National Institutes of Health funding of surgical research. Annals of Surgery 236: 277–287.

4. Johnston SC, Hauser SL (2006) Basic and clinical research: what is the most appropriate weighting in a public investment portfolio? Annals of Neurology 60: 9A–11A.

5. Istook E (1997) Research funding on major diseases is not proportionate to taxpayer's needs. Journal of NIH Research 9: 26–28.

6. Brown ML, Lipscomb J, Snyder C (2001) The burden of illness of cancer: economic cost and quality of life. Annual Review of Public Health 22: 91–113.

7. Sandler RS, Everhart JE, Donowitz M, Adams E, Cronin K, et al. (2002) The burden of selected digestive diseases in the United States. Gastroenterology 122: 1500–1511.

8. Gross CP, Anderson GF, Rowe NR (1999) The relation between funding by the National Institutes of Health and the burden of disease. New England Journal of Medicine 340: 1881–1887.

9. McKenna MT, Michaud CM, Murray CJL, Marks JS (2005) Assessing the burden of disease in the United States using disability-adjusted life years. American Journal of Preventive Medicine 28: 415–423.

10. Anderson C (1993) A new kind of earmarking. Science 260: 483.

11. Hatziandreu E, Graham JD, Stoto MA (1988) AIDS and biomedical research funding: comparative analysis. Reviews of Infectious Diseases 10: 159–167.

12. Curry CW, De AK, Ikeda RM, Thacker SB (2006) Health burden and funding at the Centers for Disease Control and Prevention. American Journal of Preventive Medicine 30: 269–276.

13. Joiner KA (2005) The not-for-profit form and translational research: Kerr revisited? Journal of Translational Medicine 3: 19.

14. Dorsey ER, Thompson JP, Carrasco M, de Roulet J, Vitticore P, et al. (2009) Financing of U.S. biomedical research and new drug approvals across therapeutic areas. PLoS ONE 4: e7015.

15. Crow MM (2011) Time to rethink the NIH. Nature 471: 569–571.

16. Teitelbaum MS (2008) Structural disequilibria in biomedical research. Science 321: 644–645.

17. Tallon D, Chard J, Dieppe P (2000) Relation between agendas of the research community and the research consumer. Lancet 355: 2037–2040.

18. Varmus H (1999) Evaluating the burden of disease and spending the research dollars of the National Institutes of Health. New England Journal of Medicine 340: 1914–1915.

19. Buxton M, Hanney S, Jones T (2004) Estimating the economic value to societies of the impact of health research: a critical review. Bulletin of the World Health Organization 82: 733–739.

20. Murphy K, Topel R (2003) Diminishing returns? The costs and benefits of improving health. Perspectives in Biology and Medicine 43: S108–S128.

21. Johnston SC, Rootenberg JD, Katrak S, Smith WS, Elkins JS (2006) Effect of a US national institutes of health programme of clinical trials on public health and costs. Lancet 367: 1319–1327.

22. Cutler DM, McClellan M (2001) Is technological change in medicine worth it? Health Affairs 20: 11–29.

23. Wooding S, Hanney S, Buxton M, Grant J (2005) Payback arising from research funding: evaluation of the arthritis research campaign. Rheumatology (Oxford, England) 44: 1145–1156.

24. Fleurence RL, Torgerson DJ (2004) Setting priorities for research. Health Policy 69: 1–10.

25. Markowitz HM (1952) Portfolio selection. Journal of Finance 7: 77–91.

26. McFarlane FW (1981) Portfolio approach to information systems. Harvard Business Review 59: 142–150.

27. Chandra S (2003) Regional economy size and the growth/instability frontier: Evidence from Europe. Journal of Regional Science 43: 95–122.

28. Birch S, Gafni A (1992) Cost effectiveness/utility analyses. Do current decision rules lead us to where we want to be? Journal of Health Economics 11: 279–296.

29. O'Brien BJ, Sculpher MJ (2000) Building uncertainty into cost-effectiveness rankings: Portfolio risk-return tradeoffs and implications for decision rules. Medical Care 38: 460–468.

30. Bridges JFB, Stewart M, King MT, van Gool K (2002) Adapting portfolio theory for the evaluation of multiple investments in health with a multiplicative extension for treatment synergies. European Journal of Health Economics 3: 47–53.

31. Bridges JF, Terris DD (2004) Portfolio evaluation of health programs: A reply to Sendi *et al.* Social Science & Medicine 58: 1849–1851.

32. Sendi P, Al MJ, Gafni A, Birch S (2003) Optimizing a portfolio of health care programs in the presence of uncertainty and constrained resources. Social Science & Medicine 57: 2207–2215.

33. Sendi P, Al MJ, Rutten FFH (2004) Portfolio theory and cost-effectiveness analysis: A further discussion. Value In Health 7: 595–601.

34. Porter ME (2010) What is value in health care? New England Journal of Medicine 363: 2477–2481.

35. Lane J, Bertuzzi S (2011) Measuring the results of science investments. Science 331: 678–680.

36. Black N (2009) Health services research: the gradual encroachment of ideas. Journal of Health Services Research & Policy 14: 120–123.

37. Prieto L, Sacristan JA (2003) Problems and solutions in calculating quality-adjusted life years (QALYs). Health and Quality of Life Outcomes 1: 80–87.

38. Burckhardt CS, Anderson KL (2003) The quality of life scale (QOLS): Reliability, validity, and utilization. Health and Quality of Life Outcomes 1: 60–66.

39. Couzin J, Miller G (2007) Boom and bust. Science 316: 356–361.

40. Freeman R, Reenen JV (2009) What if Congress doubled R&D spending on the physical sciences? Technical Report 931, Center for Economic Performance.

41. Merton RC (1972) An analytic derivation of the efficient portfolio frontier. Journal of Financial and Quantitative Analysis 7: 1851–1872.

42. Grant M, Boyd S (2009). CVX: Matlab software for disciplined convex programming. URL `http://stanford.edu/~boyd/cvx`.

43. Grant M, Boyd S (2008) Graph implementations for nonsmooth convex programs. In: Blondel V, Boyd S, Kimura H, editors, Recent Advances in Learning and Control (a tribute to M. Vidyasagar), Berlin / Heidelberg: Springer, Lecture Notes in Control and Information Sciences. pp. 95–110.

44. Bawa V, Brown S, Klein R (1979) Estimation Risk and Optimal Portfolio Choice. Amsterdam: North-Holland.

45. Cremers J, Kritzman M, Page S (2003) Portfolio formation with higher moments and plausible utility, technical report. Technical Report 272–12, Revere Street Working Paper Series.

46. Black F, Litterman R (1992) Global portfolio optimization. Financial Analysts Journal 48: 28–43.

47. Bade A, Frahm G, Jaekel U (2009) A general approach to Bayesian portfolio optimization. Mathematical Methods of Operations Research 70: 337–356.
